# Cine acquisition strategies for visualizing atrial septal defects by CMR

**DOI:** 10.1186/1532-429X-15-S1-P286

**Published:** 2013-01-30

**Authors:** Arun Natarajan, Evangelia Nyktari, Arun J Baksi, Heiko E Kindler, Philip J Kilner

**Affiliations:** 1 CMR Unit, Royal Brompton Hospital, London, UK, London, U K

## Background

Atrial septal defects (ASDs) may escape detection before adulthood, particularly those in unusual locations, which may elude echocardiographic visualisation. CMR is established for the quantification of shunt flow and, if appropriately acquired, can provide clear visualisation of ASDs and adjacent structures to inform decisions regarding intervention. Our objective is to recommend cardiovascular magnetic resonance (CMR) cine acquisition strategies suitable for the visualisation of the less common as well as the common types of ASD.

## Methods

In the CMR Unit of a tertiary referral centre for adults with congenital heart disease we retrospectively reviewed the CMRs of patients with unoperated ASDs over a 3 year period to assess the suitability of cine acquisition strategies for the visualisation of different types of ASD and any associated anomalies of pulmonary venous connection.

## Results

157 patients with unoperated ASDs had CMR studies in 3 years. If already suspected, we had routinely acquired an ‘atrial stack' of cines, meaning a contiguous stack, 5mm thick, parallel to the routine ventricular short axis (SA) stack, stepping backward from the basal ventricular plane until the superior vena cava was identifiable. This orientation visualized ostium secundum ASDs well (n=117), and was also good for inferior sinus venosus defects (n=2) and unroofed coronary sinus (n=3). In superior sinus venosus defects (n=21), a transaxial cine stack was found to give clearer visualisation of both the defect and any associated anomalous pulmonary vein connection(s) (n=20, plus 4 anomalous connections identified with secundum ASDs). The transaxial cine orientation was also the more suitable one for atrio-ventricular septal defects (AVSD, n=15) as it depicted insertions of the A-V valve leaflets adjacent to the defect(s).

## Conclusions

In patients with suspected ASDs, we recommend the acquisition of an atrial SA cine stack and a transaxial cine stack that covers atrial to aortic arch levels. Additional oblique cines aligned with the defect can supplement these, as indicated in the table.

**Table 1:** Cine orientations for visualizing atrial septal defects and anomalous pulmonary veins.

	Atrial short axis stack	Transaxial stack	Additional oblique planes
Ostium secundum defect	Good	Satisfactory	4-chamber cine
Superior sinus venosus defect	Satisfactory	Good	3-chamber cine
Atrioventricular septal defect	Suboptimal	Good	4-chamber cine
Unroofed coronary sinus	Good	Suboptimal	Consider whether the coronary sinus roof is visible in all long axis cines
Inferior sinus venosus defect	Good	Suboptimal	Oblique sagittal cine
Anomalous pulmonary veins	Suboptimal	Good	Oblique coronal cine

## Funding

None

